# Driving Behaviors in Iran: Comparison of Impulsivity, Attentional Bias, and Decision-Making Styles in Safe and High-Risk Drivers

**DOI:** 10.18502/ijps.v15i4.4297

**Published:** 2020-10

**Authors:** Fatemeh Barati, Abas Pourshahbaz, Masode Nosratabadi, Yasaman Shiasy

**Affiliations:** 1 Department of Clinical Psychology, University of Social Welfare and Rehabilitation Sciences, Tehran, Iran.; 2 Research Unit, Paarand Specialized Center for Human Enhancement, Tehran, Iran.

**Keywords:** *Attentional Bias*, *Decision-Making*, *Impulsivity*, *Risky Behavior*

## Abstract

**Objective:** Road traffic injuries are leading cause of death and economic losses, particularly in developing countries such as Iran. Thus, increased understanding of the causes of traffic accidents can help solve this problem.

The primary goal of this study was to examine attentional bias, decision-making styles, and impulsiveness in drivers with safe or risky driving behaviors. The secondary purpose was to determine the variance of each variable among 2 groups of drivers.

**Method** **:** This was a cross sectional design study, in which 120 male drivers aged 20-30 years (60 males with risky driving behaviors and 60 with safe driving behaviors) were recruited from Tehran using sampling technique. Barratt Impulsiveness Scale (BIS), Decision-Making Style Scale (DMSQ), Manchester Driver Behavior Questionnaire (MDBQ), Self-Assessment Manikin Scale (SAM), and Dot Probe Task were used. The analyses were performed using IBM SPSS version 22.

**Results: **The mean age of participants was 26 years. Significant differences were found between impulsiveness (attentional, motor, and non planning impulsiveness) and decision-making styles (spontaneous and avoidant) between the 2 groups. Also, based on the results of discriminant function analysis (DFS), the subscales of impulsiveness and 2 decision-making styles explained 25% of the variance in the 2 groups of risky and safe drivers.

**Conclusion: **Findings of this study indicated that impulsiveness and 2 decision-making styles were predominant factors. Therefore, not only is there a need for research to reduce traffic accidents, but studies can also be helpful in issuing driving licenses to individuals.

Road traffic injuries are an important cause of physical, psychological, and financial injuries and are a major threat to the public health (1). Road traffic injuries are the leading cause of death in those aged 4-34 years. Because road traffic injuries mostly affect young people, they impose a considerable financial burden on the society (2). Road accidents were the ninth leading cause of global deaths in 2004, causing 1.3 million deaths per year, and they are expected to become the fifth cause of death by 2030 (3). In Iran as a developing country around 800 000 people are injured and 19 000 die in road accident each year (4). Numerous studies have been conducted on the human factors involved in this road traffic injuries (5-7). About 60% of road traffic injuries are caused by risky driving behaviors (8).

The role of psychological factors is important in accidents. Recent studies have paid a special attention to the role of cognitive factors, including impulsiveness, attentional bias, and decision-making styles in driving behaviors and have found significant associations between these factors and risky driving behaviors (9-12). The important role of personality traits have also been shown by previous studies (13, 14). 

Multiple studies have shown that impulsive personality has a major role in risky driving behaviors (5, 7), because people with a high impulsivity have problem controlling their behavior (15). Impulsivity is a type of action without thinking about or properly evaluating the consequences (16). 

In other words, impulsiveness refers to a tendency to act suddenly without any planning in advance according to external or internal stimuli, without considering the personal or social outcomes of the action (17).

Barratt et al (1997) designed a comprehensive systematic theory involving biological, behavioral, environmental, and cognitive factors. They distinguished between 3 aspects of impulsiveness: (1) motor impulsiveness (tendency to act with stubbornness and without planning in advance), (2) attentional impulsiveness (inability to focus on immediate tasks or cognitive instability), and (3) non-planning impulsiveness (inability to plan or think carefully) (18).

Decision-making is another important factor in driving behaviors. Making decisions during driving is a vital factor in driving behavior (19), and proper decision-making can reduce the risk of road traffic injuries (20). Scott and Bruce (1995) defined decision-making styles as repeated behavioral patterns shown by people when faced with situations that require decision-making (21). Several categorizations of decision-making styles exist, and one of the most important of which is the Harren’s approach involving 3 decision-making styles: rational style (making decisions based on logic), dependent style (making decisions based on beliefs and expectations of other people), and intuitive style (making decisions based on emotions). Later the avoidant style (tendency to avoid or postpone making decisions) was added to this model (19). Decision-making during driving is a vital factor in the model of driving (20).

Attentional bias is another cognitive factor with an important role in driving behavior (22). Attentional bias refers to selective focus of attention on certain aspects of a stimulus. Attention is paid selectively to maintain limited cognitive resources at the early stages of processing environmental information in a way that is compatible with personal goals. This is an automatic process that occurs outside of conscious awareness (23).

From the cognitive perspective, impulsivity, decision-making styles, and attentional bias have different meanings (24-26). For example, the cognitive perspective of impulsivity is the inability to inhibit behavioral impulses and thoughts, which play an important role in personal and social functioning (27). Also, based on prior studies, decision-making is a predominant cognition function that has effective influence on individual's behavioral inhibition (28). In fact, defective control functions as an executive function, inability to forego immediate pleasure, and impulsivity as emotional state can be a powerful predictors for risky decision-making (26). On the other hand, attentional bias can influence the occurrence of road traffic injuries (24), as most of previous studies have focused on samples in experimental conditions rather than real conditions (accidents or driving violations)(29). Therefore, the aim of this study was to evaluate the impulsivity, attentional bias, and decision-making styles among high-risk and safe drivers in real condition.

## Materials and Methods

This was a cross sectional study in which drivers aged 20-60 years who met the inclusion criteria were recruited. Sampling was done among high-risk drivers. According to the literature review, the prevalence of high-risk driving behaviors was high among male drivers compared to females with high risk driving behavior. In this study, drivers who met the inclusion criteria for entering the high risk group, were male drivers aged 20-34 years; this was consistent with the research literature (30, 31). A total of 60 men with risky driving behaviors and 60 men with safe driving behaviors who met the inclusion criteria were selected as the study sample via a convenience sampling method. Based on the discriminant function analysis, sample size needed to be at least 20 times the number of predictive variables (32). Participants in the 2 groups were matched by age and education.

At the first stage, the researcher intended to recruit drivers from car clearance centers (storages for seized vehicles). However, sampling was not possible in the Car Clearance Center because we needed a lap top to do it, but due to security reasons we were not allowed to carry or use lap tops in the center. Therefore, sampling was done at Pasargad Insurance, Iran Insurance, and Sadeghieh Occupational Medicine center in Tehran. The initial sample included 120 participants, but due to attrition, a total of 117 participants entered the analysis. After collecting the demographic information, first, Dot Probe Task and then Barratt Impulsiveness Scale (BIS), Manchester Driver Behavior Questionnaire (MDBQ), and Decision-Making Style Scale (DMSQ) were administered. The data were analyzed with independent samples t test, repeated measures ANOVA, and discriminant function analysis. All analyses were performed using SPSS, version 22.

The following measures were taken to protect participants’ privacy: Written consent forms were obtained from all the participants, and they were allowed to leave the study at any time.

The participants could leave the study at any time.

The participants were reassured that their personal information would remain confidential and would only be used for research purposes.

Codes were substituted with real names in questionnaires and the computerized task, and only the researcher kept the information.

***Risky Driving Group***

To examine the human factors of road accidents, we should distinguish between driving errors and violations. Error is the inability to exercise proper judgment or perform a series of actions designed to achieve a desirable result, which include slips and blunders. Violations include the behaviors that increase the risk of driving. There are 2 types of violations: unintentional violations and intentional violations. Unintentional violations include unintentional behaviors that lead to violation of rules, such as driving slowly in a narrow 2-sided road. Intentional violations include behaviors aimed at harming others or violating the rules, which are considered a form of vandalism (33). Therefore, drivers with risky driving behaviors were selected from among drivers attending the Sadeghieh Center of Occupational Medicine and Pasargad and Iran insurance branches, who met the inclusion criteria. According to the research literature, high risk drivers were recruited based on following inclusion criteria (34, 35): the owners of a vehicles seized over the past year due to intentional violation, or those who were involved in accidents because of intentional violation and referred to the insurance office; derivers who had a driver's license for at least 2 years. High-risk driving behavior (high- risk probability of intentional violation) was classified based on Manchester Driving Behavior Questionnaire (MDBQ) (36). 

***Safe Driving Group***

The drivers with safe driving behaviors were matched with those with risky driving behaviors by age and education. This group included drivers who had their driving license for at least 2 years, and had no history of risky driving behaviors, including intentional violations (34).

The exclusion criteria for both groups included presence of the diagnostic criteria of an active psychiatric disorder at the time of interview and history of brain injury or epilepsy.

First, the Dot Probe Task and then the other instruments were administered for the participants. Data were analyzed using independent samples t test, repeated measures ANOVA, and discriminant function analysis. All analyses were performed using SPSS, version 22.

***Instruments***

**The Barratt Impulsiveness Scale Version 11 (BIS-11):** This 30-item scale was developed by Barratt in 1995. The items are answered on a 4-point scale, ranging from 1 (never) to 4 (always) and assess 3 factors: motor impulsiveness (acting on the spur of the moment), attentional impulsiveness (tenancy to make quick decisions), and nonplanning impulsiveness (lack of foresight) (37). Different studies have shown the psychometric properties of the BIS-11 (38, 39). Numerous studies have been conducted on the psychometric properties of the Barratt Impulsiveness Scale in both clinical and nonclinical groups. The results indicated the adequacy of the questionnaire in clinical and research settings (17). Therefore, this questionnaire was used in this study. In a study by Ekhtiari et al, which aimed at validating the Persian version of the BIS, the Cronbach’s alphas of .83 and .84 were reported in healthy people and those with substance abuse disorder, respectively (40). In the present study, we also found an alpha of .83 for the BIS. 

**The Manchester Driver Behavior Questionnaire (MDBQ):** This 50-item questionnaire was developed by Reason et al in 1990 at the University of Manchester. Validated in various European countries, the MDBQ is based on the idea that there are different psychological causes for violations and errors and that the 2 constructs should be distinguished (41). MDBQ is also commonly used to assess driving behavior and can assess aberrant driving behavior (violations and errors). The items are scored on a 5-point Likert-type scale from 0 to 5 and are of different nature based on type of driving behavior and the level of risk associated with them. Aberrant driving behavior can be divided into 4 categories: intentional violations, unintentional violations, errors, and lapses. In addition, driving behaviors are divided into 3 categories according to the level of risk associated with them: (A) behaviors that pose no risk to other drivers and do not disturb them at all (low risk‎); (B) ‎behaviors that put other drivers at a moderate level of risk (moderate risk); and (C) behaviors that surely put other drivers at risk (high risk). Various studies consider this questionnaire as a suitable instrument to measure high-risk behaviors (42). The MDBQ has acceptable validity and reliability estimates. The reliability of the MDBQ was assessed in 80 drivers via a test-retest examination with a retest interval of 8 months, and correlations of 0.75 and 0.81 were found for violations and errors, respectively (43). In Alavi et al's survey, reliability of the 2 halves of the test was 0.77(36).The validated Persian version of the questionnaire was used in the present study (36, 42), and a Cronbach’s alpha of .94 was found for the total questionnaire.

**The General Decision-Making Style Questionnaire (GDMSQ):** This 25-item questionnaire was developed by Scott and Bruce in 1995 to examine decision-making styles (21). The GDMSQ evaluates 5 different decision-making styles, including intuitive, rational, dependent, spontaneous, and avoidant. The items are rated on a 5-point scale, ranging from 1 (complete disagree) to 5 (completely agree) (44). Previous studies have shown the acceptable psychometric properties of the GDMSQ. Robert et al (2000) examined the reliability of the 5 subscales of the questionnaire and reported a range of Cronbach’s alpha from 0.62 to 0.87(45). In Iran, Moghaddam and Tehrani (2008) found Cronbach’s alphas ranging from 0.63 to 0.81 for the subscales and an alpha of 0.78 for the total GDMSQ. They also found an acceptable construct validity (46). In the present study, we found alphas of 0.71, 0.69, 0.62, 0.43, and 0.80 for the rational, avoidant, intuitive, dependent, and spontaneous styles, respectively, and an alpha of 0.64 for the total GDMSQ.

**The Dot Probe Task:** This computer-based task was developed by MacLeod (1986) using words, and it is commonly used to assess attentional bias (47). This task is usually used to assess vigilance to a specific stimulus. We used neutral pictures of risky driving as stimuli. In Iran, Dehghani et al (2010) reconstructed the Dot Probe Task using emotional faces of Iranian individuals. Dot probe tasks which assess attentional bias were developed using EFFECT software (48). 

• In the present study, the task was used with some modifications as follow:

First, the manual was shown on the monitor. Then, a + appeared on the screen for 500 milliseconds that helped the participant concentrate on the center of the screen. Following the disappearance of the plus sign, 2 other stimuli appeared on either side of the screen for 500 milliseconds and disappeared simultaneously. Then, a bright dot appeared where the former stimulus located (right or left) and stayed on the screen until the proper key on the keyboard was pressed by the participant. The reaction time to the bright dot was assessed by the computer. The bright dot took the places of the neutral and emotional stimuli equally. Slower reaction times indicated lower levels of vigilance to the presented stimuli (in the present study, pictures of risky or neutral driving) (48). The Self-Assessment Manikin Scale (SAM) was used to select pictures for the Dot Probe Task.

**Figure 1:**
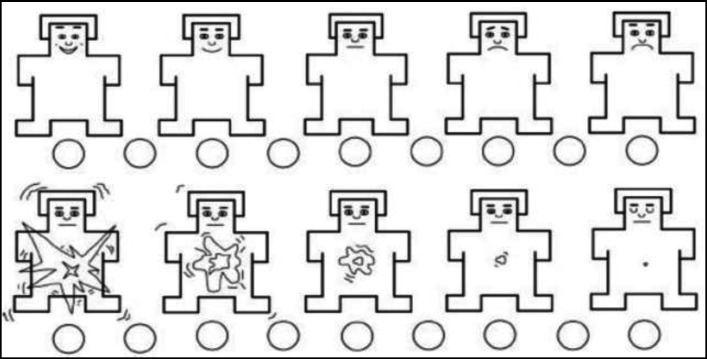
Self-Assessment Manikin Scale (SAM)

**The Self-Assessment Manikin Scale (SAM):** We used the paper-and-pencil, the 9-point version of the SAM developed by Lang in 1980 (49) to select pictures for the Dot Probe Task. The SAM consists of pictures assessing 3 elements: arousal, pleasure, and dominance. In the paper-and-pencil SAM, pleasure consists of a series of images showing “a smiling happy face” to “a frowning unhappy face.” In fact, Pleasure has pictures showing complete happiness, agreeableness, pleasure, hope, or contentment at one end of the spectrum and pictures showing complete unhappiness, depression, discontent, or hopelessness at the other end of the spectrum. Arousal has pictures showing “completely open eyes” to pictures depicting “sleepiness and calmness.” It has, in fact, pictures showing complete excitement, distress, arousal, or total awareness (open eyes) at one end of the spectrum and pictures showing complete comfort, sleepiness, calmness, inaction, or lethargy at the other end of the spectrum. Arousal and pleasure are 2 dimensions mainly used by recent studies on emotion; therefore, we excluded dominance (50). Considering that the SAM is not specific to a certain culture or language, it can be used in different cultures (51). The SAM pictures used in our study are presented in the figure (1).

**The International Affective Picture System (IAPS):** This system was used to choose neutral images. Pictures showing risky driving behavior were shown to 10 drivers from Tehran and proper pictures were selected. For each picture that was presented, the drivers were asked to determine whether it showed a risky driving behavior or not. Then, they assessed the picture on a scale using the SAM. The pictures perceived by all of the 10 drivers as showing a risky driving behavior were selected. In addition, only those pictures were selected that were related to the highest level of arousal and the lowest level of pleasure. Finally, 32 pictures, including 16 neutral pictures and 16 pictures showing risky driving behavior were selected. The resolution of the pictures was set at 200x300 pixels.

## Results

A total of 116 men participated in the present study. The mean (SD) age of participants was 26.43 years (SD = 3.87), and 69.3% of the participants were single and 30.8% were married. In addition, 44.5% of the participants did not complete high school, or had a high school diploma, or an associate degree; 41.4% had a bachelor’s degree, and 13.8% a master’s degree. Moreover, 68.4% of the participants had a full-time job, 15.4% had a part-time job, and 12.8% were students.

No significant difference was found between the 2 groups in age, marital status, education, and job status (Table 1). 

Independent samples t test was used to compare the 2 groups for attentional bias, impulsivity, and decision-making styles. No significant difference was found between the 2 groups in attentional bias and rational, intuitive, and dependent decision-making styles, but significant differences were found in attentional impulsiveness, motor impulsiveness, and nonplanning impulsiveness and spontaneous and avoidant decision-making styles (Table 2).

Repeated measures ANOVA was used to compare scores on impulsiveness and decision-making styles between drivers with safe or risky driving behaviors. In this study, independent t test was used to determine difference between groups for impulsivity and decision-making style. The repeated measure ANOVA was used to identify different forms of impulsiveness and decision-making style. Thus, the 2 groups (high-risk and safe drivers) were considered as intergroup variables while scores on decision-making style and impulsivity were assessed as an intragroup variable. Although there were 2 comparison groups, the researchers decided to use repeated measure ANOVA to minimize error. Consequently, each variable with subscales was presented and each of these subscales was considered as a level. Therefore, the 2 groups were compared at the same time based on different subscales. On the other hand, if the research team used independent t test for each subscale, they had to repeat the test for several time. Thus, repeated measure ANOVA as a strong and advanced analysis was considered.

**Diagram 1:**
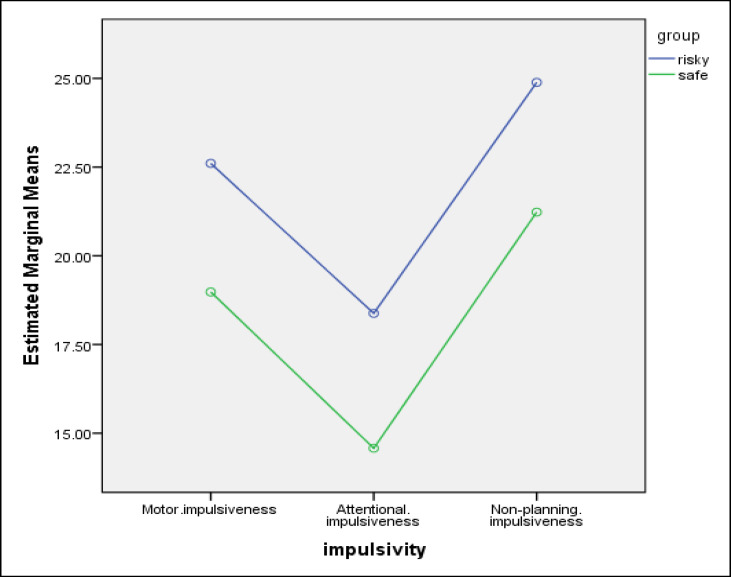
Impulsiveness Subscales’ Means for the Risky and Safe Driving Groups 2 Groups

**Diagram 2:**
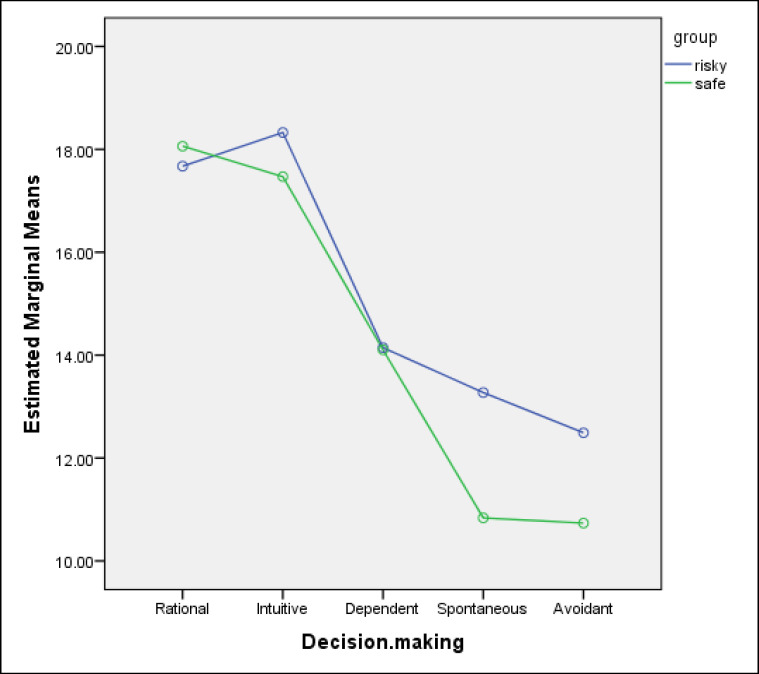
Decision-Making Styles’ Means for the Risky and Safe Driving Groups 2 Groups

 Significant differences were found in scores on the impulsiveness subscales between the 2 groups of drivers (Table 3 and Diagrams 1 and 2). In other words, drivers with risky driving behaviors scored higher on all the 3 subscales of impulsiveness (attentional, motor, and nonplanning). In addition, significant differences were observed between the 2 groups in decision-making styles. More specifically, drivers with risky driving behaviors scored higher on the intuitive decision-making style, and drivers with safe driving skills scored higher on the rational decision-making style.

Discriminant function analysis was used to determine which groups of the drivers was more similar to the study sample. Before conducting the discriminant function analysis, the assumption that the data were normal and continues was examined and confirmed. The results indicated that 25% of the variance of being a member of the 2 groups was explained by the 3 impulsiveness subscales and 2 decision-making styles. In addition, the calculated Wilks' Lambda was statistically significant (Λ = .748, p < .001).

According to the results, means of 0.564 and -0.587 were found for the drivers with risky driving behaviors and those with safe driving behaviors, respectively. Therefore, a driver with a negative score would be a member of the safe driving group and a driver with a positive score would be a member of the risky driving group.

According to the results, among the drivers with risky driving behaviors, 67.2% were correctly identified (true positive) and 32.8% were wrongly identified as drivers with safe driving behaviors (false positive). In addition, among the drivers with safe driving behaviors, 71.2% were correctly identified as drivers with safe driving behaviors (true negative) and 28.8% were wrongly identified as drivers with risky driving behaviors (false negative). The discriminant power was equal to .70. In other words, the discriminant function classified 70% of the cases correctly.

**Table 1:** Comparing Drivers with Safe Driving Behaviors with Those with Risky Driving Behaviors in Demographic Variables

**Demographic Variables**		**Risky Driving**	**Safe Driving**	**Sig.**
Age (years)		26.78 (4.31)	26.08 (3.40)	0.33
Marital Status	Single Married	45 (70.4) 13 (20.3)	36 (61) 23 (39)	0.10
Education	Below high school or High school Associate degree Bachelor’s degree Master’s degree	20 (34.5) 11 (17.2) 21 (32.8) 6 (9.4)	13 (22) 8 (13.6) 27 (45.8) 10 (16.9)	0.295
Job Status	Full-time Part-time Student	44 (68.8) 7 (10.9) 5 (6.3)	36 (61) 11 (18.6) 11 (18.6)	0.08

**Table 2:** Results of the Independent Samples T Test for Attentional Bias, Impulsivity, and Decision-Making Styles in Drivers with Safe or Risky Driving Behaviors

**Subscale** **Variable**		**Mean (SD)**		**Sig.**
		**Risky driving**	**Safe driving**	
Attentional Bias		-4.62 (24.34)	-6.89 (21.31)	0.59
Impulsiveness	Attentional	18.30 (4.10)	14.71 (.341)	0.001
	Motor	22.75 (5.06)	18.92 (3.40)	0.001
	Non-planning	24.82 (5.44)	21.17 (3.72)	0.001
Decision-making Styles	Rational	17.64 (3.82)	17.98 (3.34)	0.62
	Intuitive	18.19 (3.14)	17.50 (3.46)	0.28
	Dependent	14.17 (3.16)	13.91 (2.39)	0.61
	Avoidant	12.42 (3.49)	10.58 (3.39)	0.005
	Spontaneous	13.15 (4.03)	10.54 (3.52)	0.001

**Table 3:** Results of Repeated Measures ANOVA for Impulsiveness Subscales and Decision-Making Styles in Drivers with Safe or Risky Driving Behaviors

Source	F	Sig.	Eta
Impulsiveness (within-group) Impulsiveness × Group Between-Group	113.811 0.023 30.97	0.001 0.978 0.001	0.525 0.001 0.231
Decision-Making styles (Within-group)	87.66	0.001	0.462
Decision-Making styles × Group	3.23	0.02	0.03
Between-Group	8.58	0.004	0.07

## Discussion

The results indicted significant differences between the drivers with risky or safe driving behaviors in attentional, motor, and nonplanning impulsiveness (P < .05). In other words, drivers with risky driving behaviors had a higher mean on all the 3 subscales of impulsiveness. Previous studies have also indicated significant differences between the 2 groups of drivers in impulsiveness subscales (35). For example, in Karsazi et al's survey, a significant correlation was found between symptoms of attention deficit/ hyperactivity disorder and high-risk driving behaviors (52).

The present study revealed no significant difference among rational, intuitive, and dependent decision-making styles in the 2 groups. However, a significant relationship was found in avoidance and spontaneous decision-making style between the 2 groups. Although these results differs from some published studies (53, 54), they are broadly consistent with other studies (55, 56). This rather contradictory result may be due to different sampling technique and statistical analysis, different instrument, and age groups.

From a biological and neuropsychological perspective, impulsivity is associated with 2 basic motivational systems: behavioral inhibition system (BIS) and behavioral activation system (BAS). The BIS is responsible for controlling responses that lead to punishment and failure. On the other hand, BAS is responsible to control responses related to rewards and no punishment. Individuals with less active BIS are less likely to recognize unpleasant stimuli and evaluate them as a threat. Individuals with overactive BAS have difficulty in learning inhibitors because of strong motivation for reward. On the contrary, BIS with overactivity has high punishment-sensitivity (27). Reward sensitivity may manifest itself as a traffic rule violation, which has been presented among individuals with impulsivity. However, punishment-sensitivity may manifest itself as adaptation to the environment (57). Also, lesions of the orbitofrontal cortex (OFC) located in the lateral part of the ventromedial frontal cortex (VMF) can result in motor impulsivity. Although one can learn a movement and its consequences (reward and punishment), one is not able to control one’s behavior, and thus repeats the high-risk behavior. These individuals suffer from another type of impulsivity such as cognitive impulsivity, which can predict the likelihood of high-risk driving behaviors (58). A possible explanation for this may be that impulsivity is the inability to control behavioral impulses and thoughts. Impulsivity is an important component in executive functions and plays an important role in personal and social functions. Individuals based on behaviors learned in the family show immediate reaction to achieve what they want. Therefore, they are unable to evaluate the consequences of their reaction either for themselves or others (27).

 Numerous studies have been reported that decision-making as a major cognitive function is associated with behavioral inhibition (28). In fact, defective control function as an executive function, inability to forego immediate pleasure, and impulsivity as emotional state can be a powerful predictor for risky decision-making (26). Decisions can be influenced by a person's emotions so that positive emotions are accompanied by high problem-solving capacity, which can result in increasing rational decision-making (11). Also, high-risk behaviors are characterized by dysfunction of attentional processes. Individuals with high-risk behaviors pay attention only to affect-eliciting events (winning and losing); therefore, they are not able to learn from their mistakes (59). Moreover, decision-making difficulty in individuals with ventromedial prefrontal cortex lesions is related to blindness to the results and consequences of future actions (more immediate profit and fewer harm). According to previous results, prefrontal cortex has an important role in the decision-making process so that ventromedial or orbitofrontal cortex is in charge of assessing reward punishment of stimuli (60). In the context of attentional bias, when negative emotional words were presented to participants (high risk driving behaviors), response time would be longer in the Stroop test (24). A possible explanation for this result may be due to paying more attention to negative words compared to neutral words. Thus, individuals with longer reaction times in the Stroop task committed more driving violations (61). Finally, the present study showed no significant difference in attentional bias between the 2 groups, which was inconsistent with the findings of previous surveys (10, 22). The cause of this discrepancy can be attributed to dot-probe words, Stroop test, different sampling techniques, and various statistical analysis. However, in our study, dot-probe task included pictures of high-risk and safe driving was used among high-risk and safe drivers.

## Limitation

A number of limitations could have influenced the results of this study. First, sampling was not performed at car clearance centers because of the nature of the research and measurement of attentional bias by personal laptop. Second, accidents and driving violations is a multidimensional issue. Then, controlling nonhuman factors (vehicle type, weather conditions, and road conditions) and other human factors such as physical health was not possible. Third, the research team could not find high-risk women drivers, so men drivers were selected. However, as the number of women drivers is increasing, future studies should be conducted on driving behaviors of both sexes.

## Conclusion

In brief, the difference between impulsivity and decision-making styles between groups was explained by drivers' cognitive ability and increased frequency of high-risk driving behaviors. Direct or indirect effect of psychological factors on driving has led to unprincipled, low-quality and dangerous driving. All individuals who has obtained a driver's license are not qualified to drive and more psychological assessments should be performed on drivers to ensure their safe driving.
